# Plexin-B2 Negatively Regulates Macrophage Motility, Rac, and Cdc42 Activation

**DOI:** 10.1371/journal.pone.0024795

**Published:** 2011-09-23

**Authors:** Kelly E. Roney, Brian P. O'Connor, Haitao Wen, Eda K. Holl, Elizabeth H. Guthrie, Beckley K. Davis, Stephen W. Jones, Sushmita Jha, Lisa Sharek, Rafael Garcia-Mata, James E. Bear, Jenny P.-Y. Ting

**Affiliations:** 1 Department of Microbiology and Immunology, University of North Carolina at Chapel Hill, Chapel Hill, North Carolina, United States of America; 2 Lineberger Comprehensive Cancer Center, University of North Carolina at Chapel Hill, Chapel Hill, North Carolina, United States of America; 3 Integrated Department of Immunology, Center for Genes, Environment and Health, National Jewish Health, Denver, Colorado, United States of America; 4 Department of Cell and Developmental Biology, University of North Carolina at Chapel Hill, Chapel Hill, North Carolina, United States of America; Chinese University of Hong Kong, Hong Kong

## Abstract

Plexins are cell surface receptors widely studied in the nervous system, where they mediate migration and morphogenesis though the Rho family of small GTPases. More recently, plexins have been implicated in immune processes including cell-cell interaction, immune activation, migration, and cytokine production. Plexin-B2 facilitates ligand induced cell guidance and migration in the nervous system, and induces cytoskeletal changes in overexpression assays through RhoGTPase. The function of Plexin-B2 in the immune system is unknown. This report shows that Plexin-B2 is highly expressed on cells of the innate immune system in the mouse, including macrophages, conventional dendritic cells, and plasmacytoid dendritic cells. However, Plexin-B2 does not appear to regulate the production of proinflammatory cytokines, phagocytosis of a variety of targets, or directional migration towards chemoattractants or extracellular matrix in mouse macrophages. Instead, *Plxnb2^−/−^* macrophages have greater cellular motility than wild type in the unstimulated state that is accompanied by more active, GTP-bound Rac and Cdc42. Additionally, *Plxnb2^−/−^* macrophages demonstrate faster *in vitro* wound closure activity. Studies have shown that a closely related family member, Plexin-B1, binds to active Rac and sequesters it from downstream signaling. The interaction of Plexin-B2 with Rac has only been previously confirmed in yeast and bacterial overexpression assays. The data presented here show that Plexin-B2 functions in mouse macrophages as a negative regulator of the GTPases Rac and Cdc42 and as a negative regulator of basal cell motility and wound healing.

## Introduction

The plexins are a family of nine transmembrane proteins that are grouped by homology into four subfamilies: A, B, C, and D [1]. All family members share an extracellular semaphorin domain and an intracellular plexin domain-containing tail that can mediate intracellular signaling. The plexins were originally identified in the nervous system [2], [3], where they have been found to mediate diverse cell processes including axon guidance, neurogenesis, cell migration, cell proliferation and death. Plexins have also been found to function in other body systems including the reproductive, circulatory, endocrine, urinary, digestive, and immune system [reviewed in 4], [5], [6]. Similar to other plexins, the B family of plexins were originally found in the nervous system [1], and were later identified in the circulatory, endocrine, reproductive, urinary, digestive, respiratory, and immune systems [7]–[15]. The B plexin family is distinct from the A, C, and D plexins in the domains found in the intracellular tail. Two of the B family members, Plexin-B1 and Plexin-B2, contain an intracellular domain with a PDZ motif [post synaptic density protein (PSD95), Drosophila disc large tumor suppressor (DlgA), and zonula occludens-1 protein (zo-1)], which can relay extracellular signals to intracellular motifs [16]–[21].

In contrast to a paucity of studies on Plexin-B2, Plexin-B1 has been found in the immune system where it mediates processes similar to its function in the nervous system. Plexin-B1 is required for the optimal migration of monocytes and dendritic cells and proliferation and survival of B cells [22], [23]. The mechanisms mediating these effects of Plexin-B1 in the immune system are unknown, but in other cell types the phenotypic effects of Plexin-B1 have been attributed to its role as a regulator of the Rho family of GTPases [18], [19], [21], [24]–[26]. The Rho family of GTPases functions to regulate actin dynamics [27], [28]. Plexin-B1 has been shown to regulate Rho upon stimulation by binding to PDZ-Rho and LARGE [18], [19], [21], [24]. Plexin-B1, as well as Drosophila Plexin-B, have been shown to bind directly to the active GTP-bound form of the GTPase Rac but not the inactive, GDP bound form [24], [29], [30]. The downstream consequences of B family plexins binding to active Rac are not completely understood. In overexpression studies performed in HEK293 cells, Plexin-B1- Rac-GTP binding has been shown to sequester active Rac from its downstream effector p-21-activated kinase (PAK), which leads to increased cell surface expression of Plexin-B1 [30]. In Drosophila neurons, which have only one B family plexin, Plexin B binds to and down regulates Rac through an unknown mechanism [29].

Plexin-B2, the focus of our work, has been studied much less than Plexin-B1. In overexpression studies Plexin-B2 has been found to regulate the GTPase Rho [16]. When Plexin-B2 is synthetically stimulated by replacement of its extracellular domain, the intracellular PDZ domain of Plexin-B2 binds to the PDZ domain of the RhoGEFs (guanine nucleotide exchange factors) PDZ-RhoGEF and LARGE (leukemia associated RhoGEF), leading to the activation of Rho and the formation of stress fibers in fibroblast [16]. Studies of Plexin-B2 in the mouse nervous system have demonstrated that Plexin-B2 is required for normal development during embryogenesis. *Plxnb2^−/−^* embryo brains have defects in cortical patterning and in cell guidance of several cell types, resulting in neural tube closure defects and exencephaly [31], [32]. In the immune system, the function of Plexin-B2 has not been delineated, although Plexin-B2 has been identified on B cells as a marker of T dependent germinal center formation and on an undefined population of CD11b^+^ cells in the mouse liver [7], [33]. The function of Plexin-B2 on these cells types has not been reported.

We examined the expression and function of Plexin-B2 in the immune system and observed high Plexin-B2 expression in macrophages, conventional dendritic cells (cDC), and plasmacytoid dendritic cells (pDCs). To explore the effects Plexin-B2 on the immune system, we created fetal liver chimeric mice to reconstitute the immune system of wild type mice with *Plxnb2^−/−^* cells because the *Plxnb2^−/−^* mice do not survive post-partum. Reconstitution of the immune system with *Plxnb2^−/−^* cells has similar efficiency as reconstitution with wild type cells providing a feasible system for this study. The results show that *Plxnb2^−/−^* macrophages are not defective in their ability to secrete the inflammatory cytokines TNF or IL-6, or phagocytose fluorescent beads, *Escherichia coli* (E. coli), or antibody bound T cells. *Plxnb2^−/−^* macrophages are also not defective in the ability to migrate towards the recruiting cytokines stromal cell-derived factor-1 (CXCL12), macrophage colony stimulating factor (M-CSF), serum, or the extracellular matrix protein fibronectin. However, *Plxnb2^−/−^* macrophages show a significant increase in cell motility in the steady state and are capable of *in vitro* wound closure at a faster rate compared to wild type. Additionally *Plxnb2^−/−^* cells have higher levels of the active forms of the small Rho GTPases Cdc42 and Rac at the basal rate. These data show that Plexin-B2 is a negative regulator of Rac and Cdc42 and modulates cell motility in macrophages.

## Results

### Plexin-B2 is expressed in the immune system

**Figure 1:**
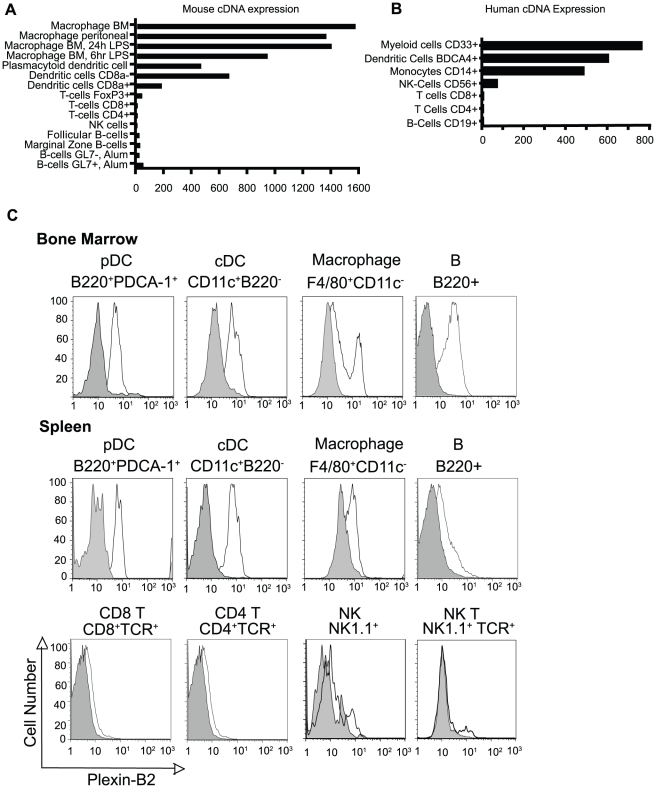
Plexin-B2 is expressed in the human and mouse immune system. cDNA expression level of *Plxnb2* in A) mouse and B) human immune cells from BioGPS database [37]. C) Plexin-B2 protein expression by flow cytometry of *ex-vivo* mouse spleen and bone marrow cells. pDCs were gated on B220^+^ PDCA-1^+^ cells. cDCs were gated on CD11c^+^ B220^−^ cells. Macrophages were gated on F4/80^+^ CD11c^−^ cells. B cells were gated on B220^+^ cells. T cells were gated on TCR^+^ cells. NK cells were gated on NK1.1^+^ TCR^−^ cells, and NK T cells on NK1.1^+^TCR^+^ cells. Wild type fetal liver reconstituted mice stain positive with anti-Plexin-B2 antibody in cells that express Plexin-B2 and are indicated by unfilled histograms. *Plxnb2^−/−^* reconstituted mice do not stain positive for Plexin-B2 and are indicated by filled histograms. Results are representative of at least four experiments, n = 8 per group.

We and others have previously shown that Plexin-A1 is found on dendritic cells in the mouse immune system and is required for optimal stimulation of T cells and for proper formation of T cell-dendritic cell conjugates by influencing actin polarization [5], [34]–[36]. These results prompted us to ask if other plexins were expressed in the immune system. *In silico* analysis of the BioGPS database [37] demonstrates that human and mouse Plexin-B2 is highly expressed on macrophages, cDCs, and pDCs, with much less to little found on B and T cells (Fig. 1A, B). This expression pattern was analyzed in the wild type (*Plxnb2^+/+^*) and Plexin-B2 deficient (*Plxnb2^−/−^*) reconstituted mice at the protein level by flow cytometry (Fig. 1C), with the reconstituted wild type serving as a negative control. Plexin-B2 is highly expressed on B220^+^ PDCA-1^+^ pDCs and CD11c^+^ B220^−^ cDCs in spleen and bone marrow. Plexin-B2 is also expressed moderately on splenic macrophages and higher on F4/80^+^ macrophages. In addition bone marrow F4/80^+^ cells show two populations based on Plexin-B2 expression. Bone marrow-derived B220^+^ B cells also express a higher amount of Plexin-B2 than splenic B cells. The low levels of B cell Plexin-B2 transcript detected by *in silico* studies (Fig. 1A, B) and the much higher protein expression (Fig. 1C) might reflect post-transcriptional control of Plexin-B2 in B cells. Very little Plexin-B2 was detected on TCR^+^CD4^+^ T cells, TCR^+^CD8^+^ T cells, NK1.1^+^ natural killer (NK) cells, or NK1.1^+^TCR^+^ NK T cells, consistent with the *in silico* studies.

### Reconstitution of the immune system with *Plxnb2^−/−^* cells is equivalent to wild type

**Figure 2:**
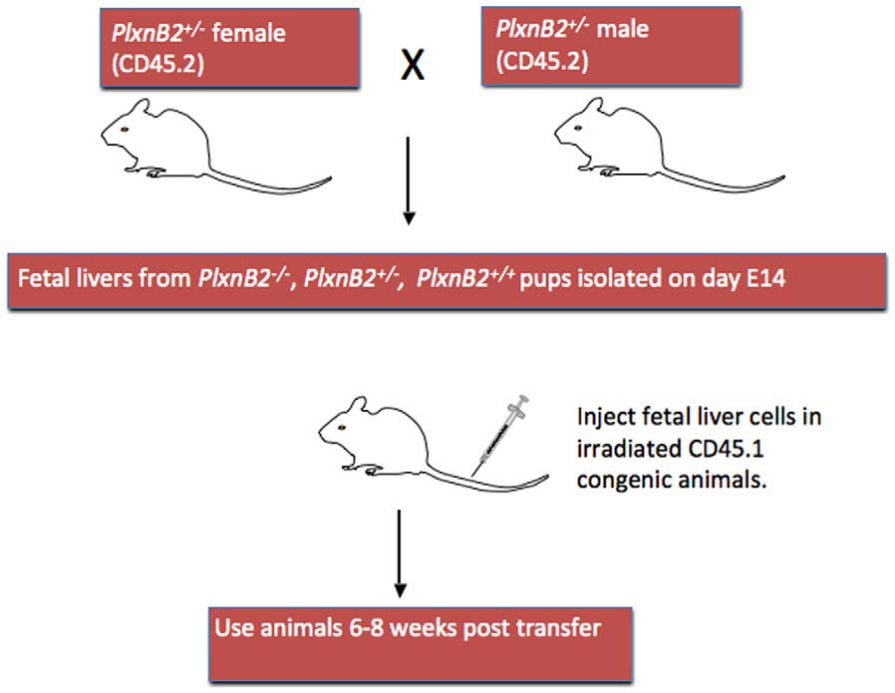
Schematic of Fetal Liver Transplant. A *Plxnb2^+/−^* male is crossed to a *Plxnb2^+/−^* female. Fetal liver cells are harvested from *Plxnb2^+/+^* or *Plxnb2^−/−^* littermate fetal livers at day 14 of gestation. 2×10^6^ fetal liver cells are injected i.v. into congenic, lethally irradiated CD45.1 recipient mice. Mice are utilized 6–8 weeks post-transplant.

**Figure 3:**
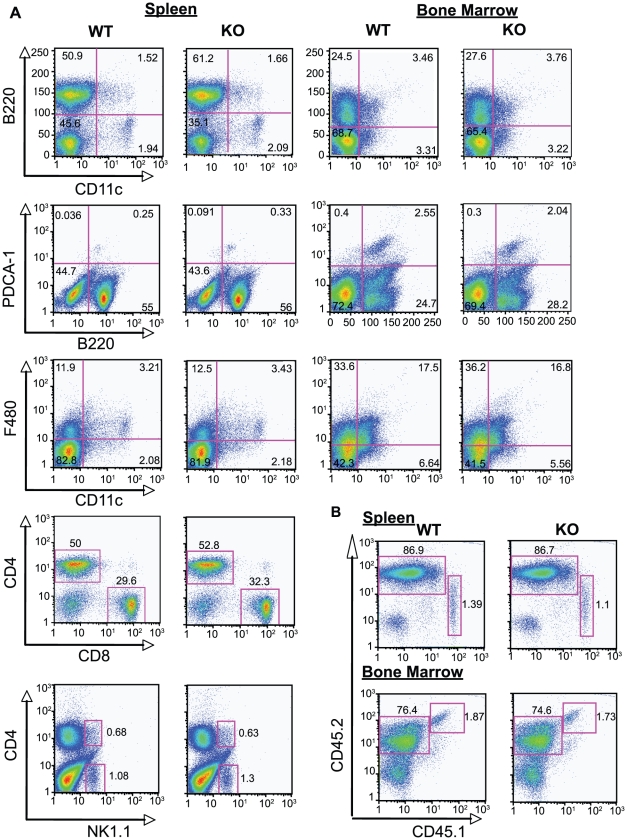
Immune system reconstitution with *Plxnb2^−/−^* fetal livers is similar to wild type. A) Flow cytometry of reconstitution of congenic CD45.1^+^ mice with wild type and *Plxnb2^−/−^* fetal livers (CD45.2^+^) in the splenic and bone marrow compartments six weeks post-transplant. cDCs are designated as B220−/CD11c+ cells, pDCs as CD11c^+^ B220^+^, and B cells as B220^+^ CD11c^−^. pDCs were further defined as CD11c^low^ and PDCA-1^+^. Macrophages were defined as F4/80^+^ cells. T cells were first gated on TCR^+^ cells, and then divided into subpopulations by the CD4^+^ and CD8^+^ markers. NK cells were defined as NK1.1+/CD4− and NKT cells as NK1.1+CD4+. B) Total donor reconstitution in the spleen and bone marrow were similar between wild type and *Plxnb2^−/−^* mice. CD45.2 marked the donor, Ly5.1^+^ cells. CD45.1 marked the residual host, Ly5.2^+^ cells. Figures are representative plots of four separate experiments. n = 5 mice per group.

Mice lacking Plexin-B2 (*Plxnb2^−/−^*) have been previously shown to have a severe phenotype, exhibiting defects in neural tube closure, cerebellar disorganization and misregulated granule cell proliferation [31], [38]. To study the immune system, it was necessary to perform fetal liver transplant from *Plxnb2^−/−^* donor backcrossed onto a C57BL/6 background for at least ten generations into C57BL/6 CD45.1^+^ recipients (Fig. 2). We and others have shown that plexins can affect cell proliferation and homeostasis [36], [39]–[43]. Therefore we studied the ability of *Plxnb2^−/−^* cells to reconstitute the mouse immune system. In the spleen and bone marrow the total number and the reconstituted percentage of B220^+^ CD11c^−^ (cDC), CD11c^low^ PDCA-1^+^ (pDC) and F4/80^+^ (macrophage) cells, which express high levels of Plexin-B2, are equivalent in recipients of wild type and *Plxnb2^−/−^* fetal livers (Fig. 3A). This also holds true for B220^+^ CD11c^−^ (B), TCR^+^ CD4^+^, and TCR^+^ CD8^+^ T cell percentages in the spleen and bone marrow (Fig. 3A). Furthermore, the total percentage of reconstituting donor (CD45.2^+^) cells compared to recipient (CD45.1^+^) is comparable for recipients of either *Plxnb2^−/−^* or wild type cells (Fig. 3B). These data suggest that Plexin-B2 does not affect cell proliferation or reconstitution of the mouse immune system in the fetal liver engraftment system. Additionally, *in vitro* generated macrophages derived from *Plxnb2^+/+^ and Plxnb2* ^−/−^ reconstituted bone marrow equally express the macrophage marker F4/80 (Supplemental Fig. S1) and produce similar cell numbers, supporting our *in vivo* mouse data which suggests that Plexin-B2 does not have an effect on hematopoietic cellular reconstitution.

### Plexin-B2 does not affect cytokine response in macrophages

**Figure 4:**
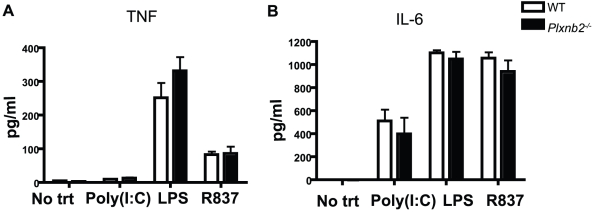
Plexin-B2 does not affect cytokine secretion. Wild type and *Plxnb2^−/−^* macrophages were plated in complete DMEM and treated for 16 hours with TLR ligands Poly(I∶C) (10 ug/ml) (TLR 3 agonist), LPS (1 ug/ml) (TLR 4 agonist), and R837 (4 ug/ml) (TLR 7 agonist). Poly(I∶C), LPS, and R837 were purchased from InvivoGen. Supernatants were collected at the 16 hour time point and assessed for secretion of A) TNF and B) IL-6 by ELISA. Graphs are representative of three independent experiments, n = 3 mice per group.

Toll-like receptors (TLRs) function as pathogen sensors on cells by recognizing pathogen-derived products, and ligation of TLRs by their agonist can initiate an inflammatory response [44]. Plexins and their semaphorin receptors have been shown to modulate cytokine secretion in response to TLRs, as described in the examples below. Plexin-A1 is required for upregulation of IFNα after TLR stimulation [45]. Plexin-A4 is required for inflammatory cytokine production in response to variety of TLR agonist [46]. Blocking antibodies to Plexin-B1 have been shown to abrogate Sema4D-induced cytokine modulation [22]. To determine if Plexin-B2 also contributes to cytokine response after TLR signaling, the inflammatory cytokines TNF and IL-6 in cell supernatants of macrophages stimulated by the TLR ligands Poly(I∶C) (polyinosinic-polycytidylic acid, TLR3 agonist), LPS (lipopolysaccharide, TLR4 agonist), and R837 (imidazoquinoline compound imiquimod, TLR7 agonist) were assessed (Fig. 4A, B) [44]. In response to TLR ligands, *Plxnb2^−/−^* and wild type macrophages secreted comparable levels of TNF and IL-6, indicating that Plexin-B2 does not play a role in the production or release of cytokines measured in this study.

### Plexin-B2 negatively regulates cell motility in macrophages

**Figure 5:**
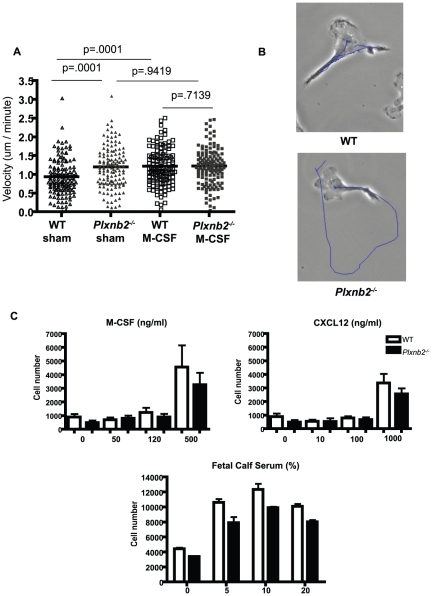
*Plxnb2^−/−^* cells have higher motility than wild type macrophages. A) Cells were plated in complete DMEM, rested overnight, and treated with sham (warmed DMEM) or warmed DMEM and 50 ug/ml M-CSF. Representative cell movement tracks over a 2.5 hour time period on sham or M-CSF treated wild type or *Plxnb2^−/−^* macrophages. B) Combined velocity scores of wild type and *Plxnb2^−/−^* bone marrow macrophages in sham or treated (50 ng/ml M-CSF) groups in 5A. Cell velocity was scored in 45 cells per group per experiment. The experiment was repeated three separate times using cells from different mice in each experiment. C) Macrophage transwell migration towards CSF, CXCL12, or FCS. Cells were placed in the upper chamber in plain DMEM and chemokines in plain DMEM in the lower chamber of migration plates. Cells were allowed to migrate for four hours towards different concentrations of cytokines (M-CSF, 0–500 ng/ml or CXCL12, 0–1000 ng/ml) or overnight towards serum (0–20%). Cells were quantified and normalized to a standard curve of each cell type. Graphs are representative of at least three independent experiments. n = 4 mice per group.

Plexin-B2 has been shown in the nervous system to mediate cell guidance, migration, and proliferation. These data prompted us to examine if Plexin-B2 could contribute to cell movement in macrophages. We compared the motility of *Plxnb2^−/−^* and wild type macrophages on glass bottom dishes using time-lapse microscopy. Three independent movies of *Plxnb2^−/−^* and wild type cells show that the mean velocity of *Plxnb2^−/−^* macrophages is significantly higher than wild type (Fig. 5A, B and Videos S1, S2, S3, S4). Cell morphology and size were similar between *Plxnb2^−/−^* and wild type macrophages throughout the experiments. We also stimulated macrophages with M-CSF and found that while wild type macrophage motility increased with M-CSF stimulation, *Plxnb2^−/−^* macrophage motility was unchanged when sham and M-CSF-treated samples were compared. Together these data indicate that Plexin-B2 serves as a motility brake in macrophages during steady state.

We also explored the directional migration capacity of *Plxnb2^−/−^* macrophages in transwell assays (Fig. 5C). Macrophages have been demonstrated to migrate towards the attractive cytokines M-CSF and CXCL12, and fetal bovine serum (FBS), [47]–[49]. The data show that migration of *Plxnb2^−/−^* macrophages towards M-CSF, CXCL12, and serum is normal compared to wild type. In all cases, *Plxnb2^−/−^* cells actually trend towards reduced migration but the results were not statistically significant. This suggests that while Plexin-B2 negatively regulates cell motility in steady state macrophages, directed migration towards M-CSF, CXCL12, and FBS are not impeded by the absence of Plexin-B2.

### Plexin-B2 Regulates Rac and Cdc42

**Figure 6:**
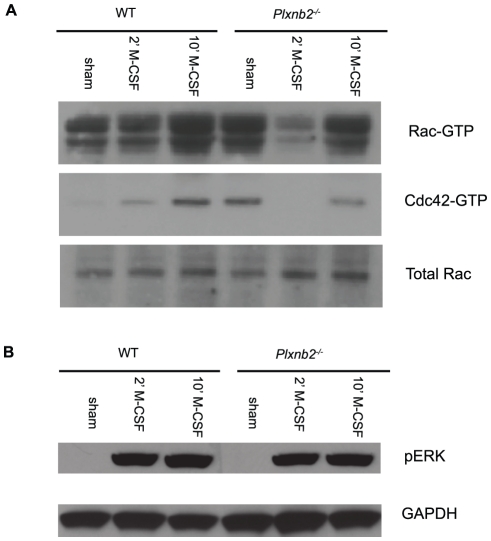
Plexin-B2 inhibits active Rac and Cdc42. WT and *Plxnb2^−/−^* macrophages were plated, rested overnight, and treated with sham (warmed DMEM) or M-CSF (50 ng/ml of M-CSF in warmed DMEM) and assessed for A) RacGTP, Cdc42GTP, total Rac and total Cdc42 using a GST-PBD pulldown assay followed by western blot. B) Western blot of ERK activation as assessed by phospho-ERK level in cells treated as above. Total protein level was determined by western blot of GAPDH. Results are representative of four separate experiments. n = 4 mice per group.

Previous studies with overexpressed Plexin-B2 in Swiss 3T3 cells have shown that Plexin-B2 binds to the active, GTP-bound form of Rac and contains a Rac binding motif in its intracellular tail [24]. Following the findings that Plexin-B2 affects cell motility, we investigated if this regulation of motility could be related to Plexin-B2 dependent regulation of Rac Rac and Cdc42 have been demonstrated to modulate cell migration and motility [28], [50], [51]. The GTP binding state of Rac and Cdc42 was assessed using GST–PBD (glutathione S-transferase PAK1 p21-binding domain) beads to pull down active Rac and Cdc42 from cell lysate followed by specific detection of each of the molecules by western blot [52], [53]. In the steady state, sham treated *Plxnb2^−/−^* macrophages repeatedly had more GTP bound Rac and Cdc42 than wild type (Fig. 6A). Following stimulation with M-CSF for two minutes, the difference was reciprocated in that wild type cells showed an increase in active Rac and Cdc42 while *Plxnb2^−/−^* cells showed a decrease in active Rac and Cdc42 (Fig. 6A). At the ten minute timepoint both wild type and *Plxnb2^−/−^* macrophages showed increased levels of active Rac and Cdc42, although the level in the WT was moderately higher (Fig. 6A). This suggests that Plexin-B2 is a negative regulator of Rac and Cdc42 when macrophages were sham treated and were in steady state, but was actually required for proper levels of Rac-GTP and Cdc42-GTP levels at the 2 minute time point after stimulation with M-CSF. The latter is in agreement with the trend of lowered migration of *Plxnb2^−/−^* macrophages in response to M-CSF, CXCL12, and FBS shown in Fig. 5. Studies with the closely related protein Plexin-B1 have shown that Plexin-B1 binding to Rac-GTP occurs in steady state without additional stimulation needed [24]. Our data shows that Plexin-B2 is similar to Plexin-B1 in that it affects steady-state Rac-GTP but additionally found that Plexin-B2 also regulates Cdc42-GTP levels. We examined the effect Plexin-B2 on the ERK (extracellular-signal-related-kinase) pathway, as previous studies of Plexin-B1 have shown that ERK is phosphorylated downstream of Plexin-B1 (Fig. 6B) [54]. Phosphorylated ERK levels in *Plxnb2^−/−^* and wild type macrophages were equivalent in response to sham treatment and in response to treatment with M-CSF at the two and ten minutes timepoints following treatment. These data represent the first evidence that the effect of Plexin-B2 in steady state cells not exposed to additional cytokine stimulation is to serve as a brake for Rac and Cdc42 activation.

### Plexin-B2 does not affect macrophage phagocytosis

The Rho family of GTPases regulate the process of phagocytosis, in which actin dynamics facilitate the cellular processes of membrane extension, phagocytic cup formation and closure, and particle uptake [reviewed in 55]. Rac and Cdc42 mediate FcγR mediated phagocytosis in macrophages [56]–[59]. Plexin-C1, in response to ligation by the viral semaphorin A39R, downregulates phagocytosis [60].

**Figure 7:**
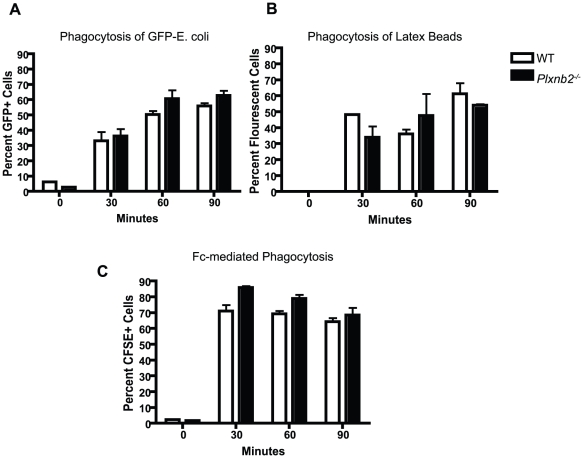
Plexin-B2 does not significantly affect phagocytosis. Wild type and *Plxnb2^−/−^* bone marrow derived macrophages were incubated with A) GFP-*E. coli*, B) latex beads, or C) opsonized thymocytes (1∶10 ratio *E. coli*, bead, or T cells: macrophage) for 30, 60, or 90 minutes. Uptake was measured by fluorescence and flow cytometry. Graphs are representative or four independent experiments. n = 5 mice per group.

To test whether the negative regulation of Rac and Cdc42 by Plexin-B2 in macrophages has an effect on phagocytosis, we performed experiments to examine the uptake of GFP-E. coli, latex beads, and antibody coated thymocytes (Fig. 7A, B, C). *Plxnb2^−/^* ^−^ or wild type macrophages were plated in complete DMEM and given either GFP-E. coli, fluorescent latex beads, or antibody coated thymocytes and assayed for cellular uptake using flow cytometry at different time points. Both *Plxnb2^−/^* ^−^ and wild type macrophages were capable of phagocytosing all treatment groups. However, the ability to phagocytose antibody coated thymocytes repeatedly trended towards greater uptake of these thymocytes by *Plxnb2^−/^* ^−^ macrophages, although this difference is not significant.

### Plexin-B2 negatively regulates wound healing

**Figure 8:**
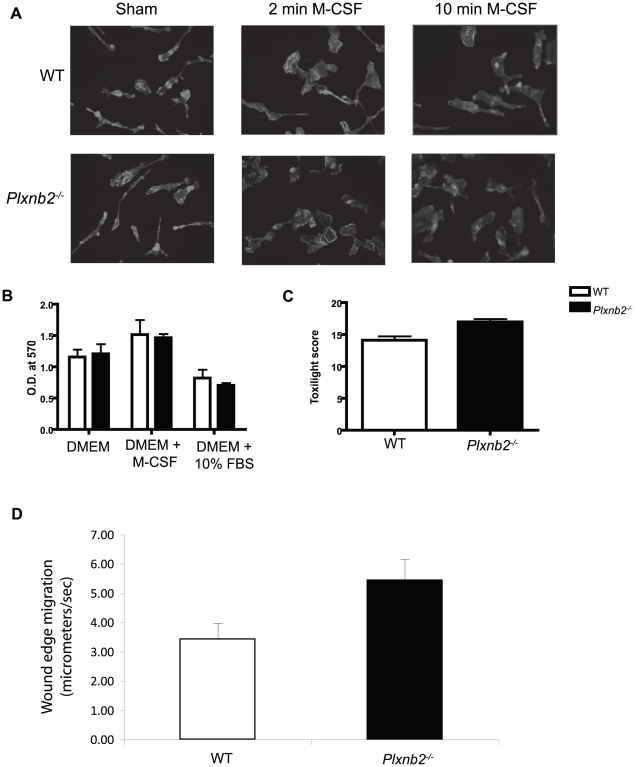
*Plxnb2^−/−^* cells demonstrate faster wound healing than wild type macrophages. A) F-actin distribution in sham and M-CSF treated macrophages. Wild type and *Plxnb2^−/−^* macrophages were plated on glass coverslips, rested overnight, and fixed following sham (plain DMEM) treatment or treatment with 50 ng/ml M-CSF in DMEM for 2 or 10 minutes. Cells were imaged on an Olympus IX-70 (Center Valley, PA). Pictures are representatives of two different experiments with 10 pictures per treatment group, with cells from four mice per group. B) Migration of macrophages into extracellular matrix protein. Wild type and *Plxnb2^−/−^* macrophages were seeded into the top chamber of a fibronectin coated transwell apparatus in plain DMEM. Cells were allowed to migrate towards plain DMEM, DMEM with 100 ng/ml M-CSF, and DMEM with 10% FBS for 20 hours and quantified by optical density. Graph is representative of two separate experiments performed with cells from four mice per group. C) Adhesion to extracellular matrix protein. Wild type and *Plxnb2^−/−^* macrophages were plated on fibronectin (20 ug/ml) coated plates and allowed to adhere for 20 minutes. Plates were washed and adherent cells were quantified by ToxiLight Bioassay Kit (Lonza, Rockland, ME). Graph is representative of two separate experiments with cells from four mice per group. D) Wound healing assay. Wild type and *Plxnb2^−/−^* macrophages (1×10^6^) were plated on 12 well plates. An artificial wound was introduced using a 200 ul pipette tip, and cells were imaged closing the wound every 10 minutes at four points per scratch for 24–48 hours using an Olympus-IX-70 inverted microscope with live cell imaging capability. The rate of change of the area of the scratch was measured over time using the NIH ImageJ program. The calculated area is in terms of pixels, and the rate of movement is calculated as pixels/sec and converted to micrometers/sec. Experiments were repeated two times with 6 wells per group, using cells from four different mice per group.

To extend the studies of the role of Plexin-B2 in functions of macrophages, we explored the possibility that *Plxnb2^−/−^* macrophages may be different from wild type in their level of F-actin, ability to bind to extracellular matrix proteins, or their ability to participate in wound-healing. To explore the effects of Plexin-B2 on the level of F-actin present in macrophages , we stained *Plxnb2^−/−^* and wild type macrophages with fluorescently labeled phalloidin, which binds to F-actin. Cells were sham (DMEM) treated or treated with 50 ng/ml of M-CSF for 2 and 10 minutes in DMEM (Fig. 8A). Macrophages of both genotypes in the sham treatment groups had similar cell morphology and actin distribution. In response to 2 minutes of treatment with M-CSF, cells from both genotypes were capable of ruffling and demonstrated similar ruffling behavior. At the 10 minute timepoint after treatment with M-CSF, both wild type and *Plxnb2^−/−^* macrophages took on the classic egg shape of the macrophage, and had similar patterns of actin staining.

To explore the role of Plexin-B2 in adhesion and migration towards extracellular matrix protein, macrophages were harvested and seeded in plain DMEM in the upper well of a 24 well fibronectin-coated transwell plate containing either DMEM, M-CSF and DMEM, or FBS (10%) and DMEM (Fig. 8B). Cells were allowed to migrate for 20 hours, and were quantified by optical density of stained cells. Both wild type and *Plxnb2^−/−^* macrophages migrated into the extracellular matrix coated transwells. We also determined the ability of *Plxnb2^−/−^* macrophages to adhere to fibronectin coated plates (Fig. 8C). Cells were plated onto fibronectin-coated 96 well plates for 20 minutes, washed, and the adherent cells were quantified by bioluminescence. *Plxnb2^−/−^* macrophages adhered to the extracellular matrix protein fibronectin in similar proportions to wild type macrophages.

Wound healing assays serve as a model of *in vitro* cell movements required to facilitate wound closure [61]. To explore the ability of *Plxnb2^−/−^* macrophages to participate in *in vitro* would healing assays (Fig. 8D and Videos S5 and S6), cells were seeded in 12 well plates in complete DMEM and rested overnight. An artificial wound in the cell monolayer was created using a pipette tip, and cells were monitored by live cell imaging. Cells were scored for wound closure over time in the image in pixels per second, and then converted to micrometers per second. *Plxnb2^−/−^* macrophages were able to move into the wound area at a rate of 5.44 pixels per second, while wild type macrophages moved into the wound area at only 3.43 pixels per second. *The Plxnb2^−/−^* macrophages closed the *in vitro* wound significantly faster (p = 0.038) than wild type macrophages, suggesting that Plexin-B2 might participate in wound healing.

## Discussion

This is the first report to explore the expression of Plexin-B2 on macrophages, cDCs, and pDCs. We found that Plexin-B2 is not expressed on resting B cells in the spleen, in agreement with previously reported expression of Plexin-B2 in B cells within T-dependent germinal centers [33]. Plexin-B2 is also not highly expressed in CD4^+^ or CD8^+^ splenic T cells, NK or NK T cells at the transcript and protein level. In the BioGPS cDNA database [37], Plexin-B2 is most highly expressed in the macrophage, cDC and pDC in the immune system. Our protein data confirms the database results. Interestingly, the same database shows Plexin-B1 has no immune system cDNA expression above median, and Plexin-B3 is most highly expressed in mast cells (data not shown). Other studies have demonstrated that Plexin-B1 is expressed in B cells, monocytes, and dendritic cells in humans [22], [23]. This suggests that expression level of different B family plexins is cell type specific and may be regulated by activation state of the cell.

The functional analyses showed a large number of functions that are unaffected by Plexin-B2, however the positive findings are that *Plxnb2^−/−^* macrophages have higher steady state motility than wild type macrophages by live cell microscopy, and perform *in vitro* wound healing at a faster rate than wild type macrophages, suggesting that Plexin-B2 is a negative regulator of cell motility under these conditions. The data demonstrates that directional migration of both *Plxnb2^−/−^* macrophages in response to cytokines M-CSF, CXCL12 or FBS are equivalent or even trended lower than wild type macrophages, suggesting that stimulated, directional migration by these stimuli is not statistically affected by Plexin-B2. The majority of the studies of Plexin B family members have relied on activation of the plexin receptor by its ligand or by synthetic dimerization. Here we show that in steady state, endogenous Plexin-B2 regulates macrophage motility. It is possible that at the steady state, Plexin-B2 may be dimerized in an autocrine fashion in our system by one of its reported ligands Sema4A, Sema4C, or Sema4D [7], [38], [62], [63]. Under this scenario, the semaphorin ligand could be secreted by macrophages, or could be provided by neighboring cells in the culture. However, we are able to use a monoclonal antibody to detect Plexin-B2 surface expression, indicating that either the anti-Plexin-B2 antibody binds to an epitope of Plexin-B2 that is not occupied by ligand binding or that Plexin B2 is not bound to its ligand in our experimental conditions.

To explore the mechanism of Plexin-B2 negative regulation of cell motility, GTP pull down assays were carried out to determine the amount of active Rac and Cdc42. The data show that in the unstimulated state, *Plxnb2^−/−^* cells have higher levels of the GTP bound, active forms of Cdc42 and Rac. Previous reports have shown that Plexin-B2 in its undimerized form binds to Rac-GTP in yeast and in bacterial overexpression studies [24]. Our data suggests that in the absence of Plexin-B2, there is more active Rac. One possibility is that Plexin-B2 might increase RacGAP (Rac GTPase accelerating protein) activity by serving as a RacGAP itself or recruits a RacGAP to the Plexin-B2 complex. However structural and biochemical studies of Plexin-B1 have shown that Plexin-B1 does not function as a RacGAP, suggesting that the Plexin-B1 modulation of Rac is through other mechanisms [64], [65]. The intracellular tails of Plexin-B1 and Plexin-B2 are similar (61% identical in their amino acid sequence – data not shown). Whether Plexin-B2 also does not function as a RacGAP requires further investigation. Studies of Plexin-B3 have demonstrated that Plexin-B3 regulates Rac activation in a ligand-dependent manner in glioma cells by binding to RhoGDI, which prevents Rac activation and reduces cell motility [66]. It is possible that at the steady state, Plexin-B2 may be interacting in a similar manner. However, upon stimulation by M-CSF, additional pathways may contribute to the regulation of Plexin-B2 and Rac. Studies of prostate cancer samples show that mutations present in the Rac binding domain of Plexin-B1 result in an increase in cell motility [67]. In our system, Plexin-B2 facilitates the downregulation of Rac and Cdc42 without the addition of ligand, suggesting that Plexin-B2 functions without ligand or that its ligand is derived autonomously. It is possible that a component in serum present in the cell culture of the macrophages may also contribute to the differences in activation state that we observed. Intriguingly, after cell stimulation by M-CSF for two minutes, levels of active Rac and Cdc42 are briefly reduced in the *Plxnb2^−/−^* cells. The biologic relevance of this is unclear.

This study revealed several functions that are not regulated by Plexin-B2. The reconstitution ratios of wild type or *Plxnb2^−/−^* fetal liver cells are similar in wild type recipients, and the percent of specific immune cell populations in the spleen and bone marrow reconstituted equivalently. This includes cell types that express the highest levels of Plexin-B2: the cDC, pDC, and macrophage. Numbers of macrophage cells derived from bone marrow cultures of *Plxnb2^−/−^* and wild type cells are equal, suggesting that Plexin-B2 does not contribute to detectable differences in immune system ontogeny or proliferation in this study. Antibody-mediated phagocytosis is a Rac and Cdc42 dependent mechanism [59]. Therefore we explored the phagocytic capacity of *Plxnb2^−/−^* macrophages compared to wild type. However, the findings show that the phagocytosis of GFP-*E. coli* and latex beads is similar for *Plxnb2^−/−^* and wild type macrophages, although, there was a trend of increased phagocytosis of antibody-coated thymocytes by *Plxnb2^−/−^* macrophages. Antibody-mediated phagocytosis has been shown to be dependant on Rac and Cdc42 [58], while bacteria bypass the need for activation of the small GTPases for cellular uptake [68]. It is possible that the over-activation of Rac and Cdc42 in the absence of Plexin-B2 leads to a modest upregulation of antibody-mediated phagocytosis, but not phagocytosis of *E. coli* and latex beads. The modest upregulation of antibody-mediated phagocytosis was consistently observed, but yet not statistically significant, suggesting that other signaling pathways important for FcR mediated phagocytosis may compensate for loss of Plexin-B2. Previous reports have shown that Plexin-B1 and other plexins can influence cytokine production in monocytic cells, suggesting the Plexin-B2 may function similarly [22], [69]. However this study shows that TNF and IL-6 production and secretion in response to TLR ligands are similar between wild type and *Plxnb2^−/−^* macrophages. Finally, we did not observe differences between wild type and *Plxnb2^−/−^* macrophages in their levels of F-actin, or in their ability to adhere or migrate to fibronectin, suggesting that Plexin-B2 does not affect F-actin levels present in macrophages, and also does not affect the ability of macrophages to adhere or migrate towards extracellular matrix proteins.

During the submission of this paper, a paper was published suggesting that Sema4C may be the ligand to Plexin-B2 in the developing kidney [70], while another paper suggests that Plexin-B2 has two ligands in the nervous system, Sema4C and Sema4G [71]. Previous literature implicated Sema4C and Sema4A as potential ligands for Plexin-B2 in non-immune cells [7], [38], [63]. Research in the field has consistently demonstrated that the issue of Plexin ligands is complex. Plexins may have multiple semaphorin ligands that vary by cell type or tissue, may bind to other types of ligands, or may bind homophilically [72]–[74]. In Fig. S2, we show that Sema4C is expressed in macrophages, however its expression pattern is strongly induced by LPS, while that of Plexin-B2 is reduced by LPS, suggesting Sema4C may not be the ligand for Plexin-B2 in macrophages. Future research will determine if Sema4C or other semaphorins serve as an autocrine or paracrine ligand of Plexin-B2 in the macrophage, and the effects of Plexin-B2 ligands on macrophage function and cytokine secretion.

In summary, this study shows that Plexin-B2 is highly expressed in the immune system on cells of monocytic-myeloid lineage, including the cDC, pDC, and macrophage. Plexin-B2 has been shown previously to bind to the activated form of Rac, but the physiological consequence of Plexin-B2 Rac-GTP binding is unknown. We show that *ex-vivo Plxnb2^−/−^* macrophages have higher levels of activated Rac and Cdc42 and increased cell motility in steady state. In addition, Plexin-B2 exerts a negative effect on an *in vitro* wound healing assay. Our data suggest that the function of Plexin-B2 in steady state is to negatively regulate Rac and Cdc42 and to maintain a brake on cell motility in steady state cells. Future research into the role of Plexin-B2 in wound healing is another area that should be of interest.

## Materials and Methods

### Ethics Statement

All studies were conducted in accordance with the National Institutes of Heath Guide for the Care and Use of Laboratory Animals and were approved by the Institutional Animal Care and Use Committee (IACUC) guidelines of the University of North Carolina at Chapel Hill (protocol #08-200).

### Mice

C57BL/6 (CD45.2) and congenic C57BL/6 (CD45.1) mice were purchased from National Cancer Institute (Boston, MA). *Plxnb2^−/−^* mice were described previously and were a kind gift from Dr. Marc Tessier-Lavigne [31]. *Plxnb2^+/−^* mice were backcrossed with C57BL/6 mice at least 10 generations at the University of North Carolina Chapel Hill. Mice were used at six to eight weeks of age and were housed in a pathogen-free barrier facility at the University of North Carolina Chapel Hill. *Plxnb2^+/−^* mice were crossed to obtain *Plxnb2^−/−^* and wild type fetal livers from stage E14 pups. Fetal liver cells were then injected intravenously into lethally irradiated C57BL/6 CD45.1 to reconstitute the immune system and analyzed 6–10 weeks post reconstitution [75].

### Cell culture

Macrophages were generated by bone marrow culture in L929 media. In brief, mouse femurs and tibias were removed from 6–8 week old mice, cleaned, and aspirated to remove bone marrow. Cells were cultured in L929 for six days. Macrophages were harvested and replated in complete DMEM media [DMEM, 10% heat inactivated FBS, non-essential amino acids, L-glutamine, sodium pyruvate, and penicillin/streptomycin (P/S)], and rested overnight before experiments.

### Reagents

Poly(I∶C), Ultrapure LPS, and Imiquimod (R837) were from InvivoGen. M-CSF was from R&D Systems (Minneapolis, MN). Antibodies for western blotting included Rac1 (C-14; sc-217), Cdc42 (B-8; sc-8401), phospho-ERK1/2 (Thr202/Thr204) (197G2; 4377) from Santa Cruz Biotechnology (Santa Cruz, CA) and GAPDH (MAB374) from Millipore (Billerica, MA). Secondary goat α mouse-HRP (horse radish peroxidase) and goat α rabbit-HRP antibodies are from Santa Cruz Biotechnology (Santa Cruz, CA). Beads for assays of GTP-bound Rac1 and Cdc42 glutathione-sepharose (GST) bead conjugated with Pak1 binding domain (Rac1 and Cdc42) were kindly provided by Dr. Keith Burridge (University of North Carolina, Chapel Hill).

### Flow cytometry

Antibodies used for flow cytometry included: B220 (RA3-6B2), CD45.2 (104), CD45.1 (A20), CD4 (L3T4), F4/80 (BM8), CD8 (Ly-2), TCR (H57-597), CD11b (M1/70), CD11c (N418), NK1.1 (NKR-P1C) PDCA-1 (BST2, CD317) and Plexin-B2 (3E7) from eBioscience (San Diego, CA). Single cell suspensions of spleen and bone marrow were lysed in ACT to remove red blood cells, washed and resuspended in FACS buffer (1× PBS and 2% FBS) at 1×10^6^ cells per well and stained with antibody combinations. All experiments were performed on a FACSCalibur (BD Biosciences, Franklin Lakes, NJ) or Cyan (Dako, Carpinteria, CA) and analyzed with FlowJo software (Tree Star, Ashland, OR).

### Live Cell Microscopy

1×10^4^ cells were plated on glass bottom dishes (MatTek Ashland, MA P35G-1.5-10-C). Cells were imaged on a Nikon Biostation (Belmont, CA) with a 20× objective. Cell velocity was analyzed using manual tracking in ImageJ [76]. Images were collected every 5 minutes for a total time of 2.5 hours for unstimulated cells or following treatment with 50 ng/ml M-CSF. For each treatment three separate movies were filmed and 45 cells scored for velocity. n = 3 mice per group.

### Migration

Macrophages were M-CSF starved in complete DMEM overnight, harvested, and seeded in plain DMEM at 2×10^5^ per upper well of 96-well transwell plates with 8 µm pores (ChemoTx System; NeuroProbe, Gaithersburg, MD), over chemokines (Peprotech, Rocky Hill, NJ) in serum-free DMEM or FBS (Gibco, Carlsbad, California) in DMEM, and incubated at 37°C for 4 hours for migration towards chemokine or overnight for migration towards serum. Migrated cells were quantified using ToxiLight Bioassay Kit (Lonza, Rockland, ME). Experiments were repeated at least three times. n = 4 mice per group. Experiments with cells migrating towards fibronectin were performed following the manufacturers' instructions (Millipore, Billerica, MA). In brief, macrophages were harvested and seeded in plain DMEM at 2×10^5^ per upper well of a 24 well fibronectin coated transwell plates containing either DMEM, M-CSF (100 ng/ml) and DMEM, or FBS (10%) and DMEM. Cells were allowed to migrate for 20 hours, and were quantified by optical density of stained cells. Experiments were repeated two times, n = 4 mice per group.

### Detection of GTP-bound Rac1, Cdc42, and pERK

Cells were plated at 2×10^6^ cells per well in six well plates and treated with 50 ng/ml M-CSF or media for the indicated times. Assays for GTP-bound Rac1 and Cdc42 were performed as described [77]. Cells were lysed and precipitated using GST-PBD beads for Rac and Cdc42. Bead bound proteins and cell lysate were resolved on a NuPAGE (Invitrogen Carlsbad, CA) gel and were transferred onto nitrocellulose membranes. Membranes were blocked with 10% milk and probed with primary antibodies against Rac1 and Cdc42 followed by appropriate secondary HRP conjugated antibody for detection. For detection of pERK cells were plated as above, treated with 50 ng/ml M-CSF for the indicated times, lysed and resolved on a NuPage (Invitrogen Carlsbad, CA) gel.

### Phagocytosis

Macrophages were plated at 1×10^5^ cells per well in a 96 well non-tissue culture treated plate in 100 ul of macrophage media without antibiotics. Cells were rested overnight and then treated with 1×10^7^ GFP *E. coli* (green fluorescent protein expressing *Eshericia coli* kindly provided by Dr. Glenn Matoshima, University of North Carolina Chapel Hill), 1×10^7^ fluorescent latex beads (Invitrogen F13080, Carlsbad, CA), or 1×10^6^ CFSE (carboxyfluorescein diacetate succinimidyl ester, Invitrogen, Carlsbad, CA) labeled, antibody labeled (CD45.2, mouse IgG2a) thymocytes as previously described [78]. Cells were washed with media, extracellular fluorescence quenched with 0.2% Trypan Blue (Sigma T8154, St. Louis, MO), fixed in 0.1% EM-grade formaldehyde and analyzed by flow cytometry for percent of fluorescent positive cells.

### ELISA

Macrophages were plated at 2×10^5^ cells per well in a 96 well plate and stimulated overnight. Cell supernatants were quantified using the ELISA kits for mouse TNF-α (555268) or IL-6 (555240) (BD Biosciences San Jose, CA).

### F-actin staining

Cells were seeded at 1×10^4^ cells in complete DMEM on glass coverslips and rested overnight. Cells were then sham treated with warmed plain DMEM, or treated with 50 ng/ml M-CSF in warmed DMEM for 2 or 10 minutes. Cells were fixed with 300 ul ice cold 4% paraformaldehyde in Krebs media for 10 minutes, washed in PBS, and permeabalized in 0.1% Triton X in PBS for 5 minutes at room temperature. Cells were blocked in 5% BSA, stained with fluorescently labeled phalloidin (Invitrogen, Carlsbad, California) and mounted. Cells were imaged at 40× using an Olympus BX-61 (Center Valley, PA).

### Adhesion to extracellular matrix protein

Well of a ninety six well plate were coated with fibronectin (20 ug/ml) overnight and washed with PBS. Cells were harvested, resuspended in media at 5×10^5^ cells/ml and incubated at 37°C without a top. Cells (50 ul) were then plated onto the fibronectin coated plate and incubated at 37°C without a lid for 20 minutes, washed 2 times with PBS. Adherent cells lysed and quantified to a standard curve using the ToxiLight Bioassay Kit (Lonza, Rockland, ME).

### Wound healing assay

Wound healing assays were performed as previously described [61]. Macrophages were seeded (1×10^6^) on 12 well plates (glass or plastic) in complete DMEM and rested overnight. An artificial wound was introduced using a 200 µl pipette tip. Cells were imaged using an Olympus IX-70 inverted microscope with live cell imaging capability every 10 minutes at four points per scratch for 24–48 hours. To calculate the rate of migration, the movies were converted to TIFF stacks and the rate of change of the area of the scratch was measured over time using NIH ImageJ program. The area calculated by ImageJ is in terms of pixels, and the rate of movement is calculated as pixels/sec and converted to micrometers/sec. Experiments were repeated two times with 6 wells per group, using cells from two different mice per group in each experiment.

### Statistical analysis

Statistical significance was determined with two-tailed Student's t test. All *p* values less than 0.05 were considered significant.

## Supporting Information

Figure S1 **Macrophage marker F4/80 is expressed by bone marrow derived wild type and** ***Plxnb2^−/−^*** **macrophages.** Flow cytometry plot of A) *Plxnb2^−/−^* and B) wild type bone marrow derived macrophages. Bone marrow cells were plated on plastic plates and grown in complete DMEM and L929 media. Cells were harvested, blocked, and stained with antibody against F4/80 and Plexin-B2 (ebiosciences, San Diego, CA). A flow cytometry plot of mixed wild type and *Plxnb2^−/−^* macrophages stained with isotype controls for F4/80 and PlexinB2 antibodies are displayed in figure C.
(EPS)

Figure S2 **Semaphorin 4C is expressed in the human and mouse immune system.** cDNA expression level of in A) mouse and B) human immune cells from BioGPS database [37].
(EPS)

Video S1 **Live cell video of cell motility of wild type macrophages.** Representative cell movement videos over a 2.5 hour time period. 1×10^4^ cells were plated on glass bottom dishes (MatTek Ashland, MA P35G-1.5-10-C) in complete DMEM, rested overnight, and treated with sham (warmed DMEM). Cells were imaged on a Nikon Biostation (Belmont, CA) with a 20× objective at ten different points. Images were collected every 5 minutes for a total time of 2.5 hours following sham treatment. Three separate movies were filmed and 45 cells scored for velocity. Cell velocity was analyzed using manual tracking in ImageJ [76]. The experiment was repeated three separate times using cells from different mice in each experiment.
(AVI)

Video S2 **Live cell video of cell motility of wild type, M-CSF treated macrophages.** Representative cell movement videos over a 2.5 hour time period. 1×10^4^ cells were plated on glass bottom dishes (MatTek Ashland, MA P35G-1.5-10-C) in complete DMEM, rested overnight, and treated with 50 ng/ml M-CSF in warmed DMEM. Cells were imaged on a Nikon Biostation (Belmont, CA) with a 20× objective at ten different points. Images were collected every 5 minutes for a total time of 2.5 hours following sham treatment. Three separate movies were filmed and 45 cells scored for velocity. Cell velocity was analyzed using manual tracking in ImageJ [76]. The experiment was repeated three separate times using cells from different mice in each experiment.
(AVI)

Video S3 **Live cell video of cell motility of** ***Plxnb2^−/−^*** **macrophages.** Representative cell movement video over a 2.5 hour time period. 1×10^4^ cells were plated on glass bottom dishes (MatTek Ashland, MA P35G-1.5-10-C) in complete DMEM, rested overnight, and treated with sham (warmed DMEM). Cells were imaged on a Nikon Biostation (Belmont, CA) with a 20× objective at ten different points. Images were collected every 5 minutes for a total time of 2.5 hours following sham treatment. Three separate movies were filmed and 45 cells scored for velocity. Cell velocity was analyzed using manual tracking in ImageJ [76]. The experiment was repeated three separate times using cells from different mice in each experiment.
(AVI)

Video S4 **Live cell video of cell motility of** ***Plxnb2^−/−^*** **, M-CSF treated macrophages.** Representative cell movement videos over a 2.5 hour time period. 1×10^4^ cells were plated on glass bottom dishes (MatTek Ashland, MA P35G-1.5-10-C) in complete DMEM, rested overnight, and treated with 50 ng/ml M-CSF in warmed DMEM. Cells were imaged on a Nikon Biostation (Belmont, CA) with a 20× objective at ten different points. Images were collected every 5 minutes for a total time of 2.5 hours following sham treatment. Three separate movies were filmed and 45 cells scored for velocity. Cell velocity was analyzed using manual tracking in ImageJ [76]. The experiment was repeated three separate times using cells from different mice in each experiment.
(AVI)

Video S5 **Live cell videos of** ***in vitro*** **wound closure of wild type macrophages.** Representative live cell imaging of wound healing assay with wild type macrophages. 1×10^6^ cells were plated on 12 well glass or plastic plates in complete DMEM and rested overnight. An artificial wound was introduced using a 200 ul pipette tip, and cells were imaged closing the wound every 10 minutes at four points per scratch for 24–48 hours using an Olympus-IX-70 inverted microscope with live cell imaging capability. To calculate the rate of migration, the movies were converted to TIFF stacks and the rate of change of the area of the scratch was measured over time using NIH ImageJ program. The area calculated by ImageJ [76] is in terms of pixels, and the rate of movement is calculated as pixels/sec and converted to micrometers/sec. Experiments were repeated two times with 6 wells per group, using cells from two different mice per group in each experiment.
(MOV)

Video S6 **Live cell videos of** ***in vitro*** **wound closure of** ***Plxnb2^−/−^*** **macrophages.** Representative live cell imaging of wound healing assay with *Plxnb2^−/−^* macrophages. 1×10^6^ cells were plated on 12 well glass or plastic plates in complete DMEM and rested overnight. An artificial wound was introduced using a 200 ul pipette tip, and cells were imaged closing the wound every 10 minutes at four points per scratch for 24–48 hours using an Olympus-IX-70 inverted microscope with live cell imaging capability. To calculate the rate of migration, the movies were converted to TIFF stacks and the rate of change of the area of the scratch was measured over time using NIH ImageJ program. The area calculated by ImageJ [76] is in terms of pixels, and the rate of movement is calculated as pixels/sec and converted to micrometers/sec. Experiments were repeated two times with 6 wells per group, using cells from two different mice per group in each experiment.
(MOV)
